# Numerical investigation of wake interaction and bio-inspired propulsive performance of tandem flapping foils for soft-robotic underwater locomotion

**DOI:** 10.1038/s41598-026-43997-5

**Published:** 2026-04-03

**Authors:** Prafulla Kumar Swain, Surya Narayan Padhi, Nidhin Raj A, Sd. Abdul Kalam, Mamata Choudhury, K. S. Raghuram, Mohammad Rafighi, Priyaranjan Sharma

**Affiliations:** 1 Department of Mechanical Engineering, GITAM Deemed to be University, Visakhapatnam, 530045 Andhra Pradesh India; 2 Department of Mechanical Engineering, Koneru Lakshmaiah Education Foundation, Vaddeswaram, 522302 Andhra Pradesh India; 3 Department of Mechanical Engineering, Adi Shankara Institute of Engineering and Technology, Ernakulam, 683574 Kerala India; 4 Department of Mechanical Engineering, PVP Siddhartha Institute of Technology, Vijayawada, 520007 Andhra Pradesh India; 5 Department of Computer Application, PSCMR College of Engineering and Technology, Krishna District, Vijayawada, 520001 Andhra Pradesh India; 6 Department of Mechanical Engineering, Vignan’s Institute of Information Technology, Visakhapatnam, 530049 Andhra Pradesh India; 7 Department of Mechanical Engineering, Başkent University, 06790 Ankara, Turkey; 8 School of Mechanical Engineering, Purdue University, West Lafayette, 47907 USA; 9 Department of Mechanical Engineering, Manipal University Jaipur, Dehmi Kalan, Jaipur, 303007 Rajasthan India

**Keywords:** Upstream foil, Downstream foil, Propulsive efficiency, Induced thrust, Tandem configuration, Tip vortex interaction, Engineering, Physics

## Abstract

This study statistically examines wake–vortex interactions and their impact on the bio-inspired propulsive performance of tandem flapping foils for soft-robotic underwater locomotion. While most existing mathematical studies emphasize tandem foils executing pure heaving, pitching, or combined motions, the present work examines four dissimilar flapping trajectories (FT-I, FT-II, FT-III, and FT-IV), including both simple and elliptical paths in single-foil and tandem configurations. Simulations are performed at a Reynolds number of 1173 to evaluate the effects of phase angle, Strouhal number (St), and flapping trajectory on thrust generation and wake interaction. The experimental findings indicate that the thrust generated by an individual flapping foil is primarily determined by the formation of vortices at the trailing edge. The mechanism works similarly to soft robotic swimmers that generate propulsion. Simple flapping mechanics produce a well-defined reverse Kármán vortex street, which generates more thrust than elliptical trajectories because it imparts more momentum to the wake. In tandem layouts, propulsive efficiency is strongly dependent on the phase difference between the upstream and downstream foils, rather than on the amplitude of flapping. The peak thrust is found at St = 0.4, especially with FT-I motion in conditions of 180-degree counter-phase and FT-III motion in conditions of in-phase operation when constructive wake interactions take place. Upstream foil operation is not very sensitive to the downstream spacing, but the downstream foil is highly sensitive to its aft location. The results provide design choices for controlling wake–vortex interactions in efficient underwater propulsion systems based on biomimetic and soft-robotic designs.

## Introduction

### Background and motivation

Tandem flapping foils have motivated many researchers worldwide to use them as effective propulsion devices^1–4^. Fish and other aquatic creatures employ a number of flapping trajectories to increase the induced force of their fins and tails. Some studies have reported in the literature to recreate the motion of aquatic animals using artificial flapping foils to enhance thrust generation, either in pure pitching^5,6^, pure heaving^7–10^, or a combination of both pitching and heaving^10–17^. These natural propulsion techniques have served as the impetus for these studies. The downstream foil can be strongly influenced by the upstream foil in a tandem-flapping setup. Thus, the propulsive action of the rear foil can be optimized by judiciously selecting design parameters, such as foil spacing, phase angle, and flapping pathway^18–21^.

Anderson et al.^14^ inferred, based on two-dimensional simulations of oscillating foils, that the primary variables determining vortex formation and thrust generation are the pitching amplitude, flapping frequency, and Strouhal number (St). Triantafyllou et al.^7^ found the same results. Lighthill^22^ established the theoretical basis for efficient aquatic propulsion, while later studies showed that the formation of a reverse Kármán vortex street^23^ is responsible for generating significant propulsive force in flapping foils. A favorable vortex interaction greatly increases hydrodynamic efficiency, according to Weihs’s^24^ investigation into the hydromechanics of fish schooling. Fish swimming in a diamond pattern needed less effort than those swimming directly behind the leader.

According to Swain et al.^25^ and Gopalskrishnan et al.^26^, when a downstream foil interacts with the incoming vortex shed by the upstream foil, the induced thrust of the downstream foil increases at lower St values but decreases at higher St values. Similar findings were obtained by Deng et al.^27^ after performing two-dimensional simulations of flapping foils traveling in sinusoidal paths. Through fish schooling experiments, Partridge & Pitcher^28^ found that hydrodynamic performance is significantly impacted by the trailing fish’s relative positioning in relation to the leader.

In contrast to swimming in still water, Liao et al.^29^ demonstrated that fish swimming behind a D-section cylinder used less energy when traveling through an existing vortex street. Beal et al.^30^ even showed that favorable flow dynamics allow a dead fish to move forward when it is placed in a vortex street. In a two-dimensional simulation of flapping foil, Shao et al.^31^ discovered that vortex energy can increase the induced thrust. In their study of vortex interactions between tandem fish models in both in-phase and counter-phase settings, Maertens et al.^32^ came to the conclusion that vortex interactions have a greater impact on downstream fish than upstream fish. Depending on the degree of vortex interaction, the downstream fish’s propulsive efficiency increased or decreased. The impact of flapping trajectory on efficiency and induced force has only been examined in a few studies. When compared to sinusoidal paths, Platzer et al.^33^ and Ashraf et al.^34^ found that non-sinusoidal flapping motions could increase thrust by up to 17%. Similar to this, Xiao et al.^35^ discovered that altering the foil’s velocity profile can result in up to 50% more thrust by introducing a trapezoidal flapping trajectory in place of the conventional heave-and-pitch motion.

Wake stability and structural flexibility have recently proven to be essential to flapping propulsion systems. It has also been demonstrated that chordwise flexibility prevents jet-switching and reinstates wake periodicity in a flapping foil, enhancing wake stability and propulsion^36^. Similarly, the vibration of flexible structures caused by the wake, as in the case of bluff bodies, makes fluid-structure interaction particularly strong and underscores the possibility of using such structures in energy-harvesting applications^37^. In addition, chaotic transitions between different wakes can be controlled with tuned chordwise flexibility, and orderly structures of vortices in flapping foils can be promoted^38^. These results suggest that wake dynamics and structural flexibility play a significant role in determining propulsion efficiency.

Recent developments in the study of tandem flapping foils have highlighted the significant role of trajectory-phase coupling in controlling hydrodynamic performance. Lin et al.^39^ demonstrated an increase in propulsive efficiency of up to 35% when phase synchronization between tandem foils is optimally tuned to the wake frequency. Yu et al.^40^ investigated the effects of elliptical and linear trajectories on vortex-street formation and found that elliptical trajectories reduce the negative pressure gradient acting on the downstream foil. Pradhan et al.^41^ reviewed the effect of inter-foil spacing on thrust generation and found that the optimal spacing is trajectory-dependent and varies with the Strouhal number. Li et al.^42^ investigated the three-dimensional effects of tandem flapping at moderate Reynolds numbers and found that tip-vortex interactions may greatly alter two-dimensional predictions. Gao et al.^43^ presented comprehensive experimental validation of phase-dependent thrust enhancement in bio-inspired tandem swimmers. Zarruk et al.^44^ investigated the structural coupling of flexible tandem foils and showed that flexibility can either improve or decrease performance based on the phase relationship. Collectively, these studies highlight the complex interplay among trajectory design, vortex dynamics, and phase synchronization in tandem configurations, motivating the present investigation of four distinct flapping trajectories under varying phase conditions.

Previous studies have analyzed the propulsion characteristics of flapping foils in simple pitching and heaving motions, most often singly or in combination. Changes in interaction and the enhancements of the vortex and thrust of upstream and downstream foils in a tandem system due to different flapping and phase differences have not been fully investigated. The current study addresses this research gap by numerically investigating four flapping-trajectory types (FT-I, FT-II, FT-III, and FT-IV) involving simple and elliptical motions at a Reynolds number of 1173. The purpose of the study is to understand how the flapping path, the phase angle (0° and 180°), and the Strouhal number (St) affect the vortex dynamics, the wake structure, and the induced thrust of the single- and tandem-foil configurations. The relationship between upstream and downstream vortices that govern thrust release and propulsive efficiency is specifically analyzed. To enhance downstream propulsion through an advantageous tip-vortex interaction and support the development of effective biomimetic propulsion systems, the analysis aims to identify the optimal combination of phase difference and trajectory type.

## Foil kinematics and computational model

### Foil kinematics

The main parameters considered in the current simulation are presented in this section. A thinner NACA 0015 (National Advisory Committee for Aeronautics) foil is chosen for both 2-D and 3-D simulations in accordance with Swain et al.‘s research^5,25^. The NACA0015 airfoil was selected for this study based on its biological relevance, as the 15% thickness-to-chord ratio closely approximates the cross-sectional geometry of fish fins and marine mammal flippers, providing a realistic basis for bio-inspired propulsion studies. NACA0015 has been widely adopted in flapping-foil research, enabling direct comparisons and validation against well-established experimental and numerical datasets reported in the literature^45,46^. Owing to its symmetric, zero-camber geometry, camber-induced bias in lift and thrust generation is eliminated, enabling isolation of kinematic and vortex-interaction effects. Furthermore, at Reynolds numbers of Re ≈ 10³–10⁴, the NACA0015 airfoil exhibits favorable boundary-layer behavior and delayed stall, making it particularly suitable for low-speed aquatic propulsion and soft-robotic underwater vehicle applications.

**Fig. 1:**
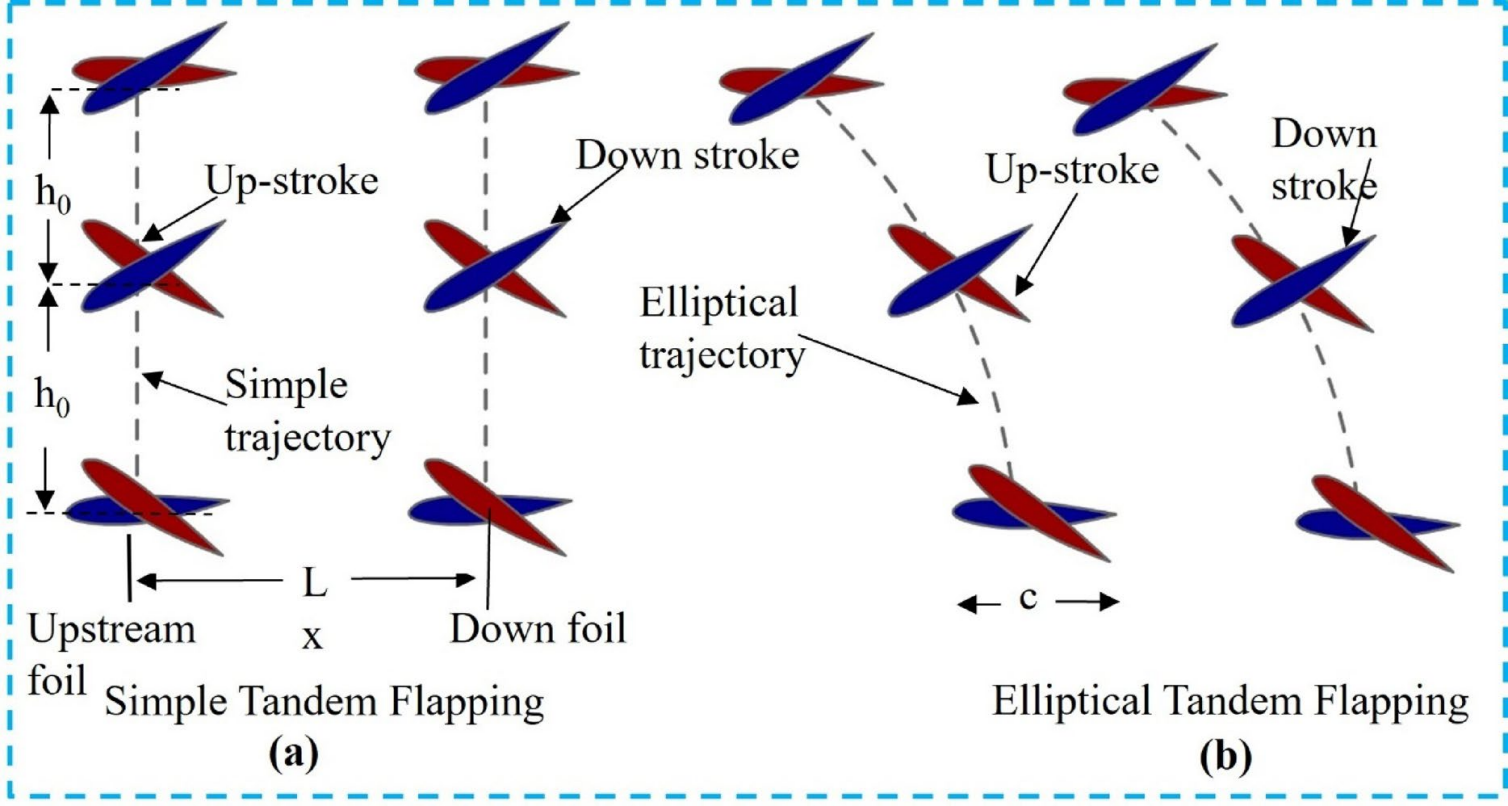
(**a**) Simple flapping motion in a tandem setup (FT-I), (**b**) Elliptical flapping motion in a tandem setup (FT-II).

**Fig. 2:**
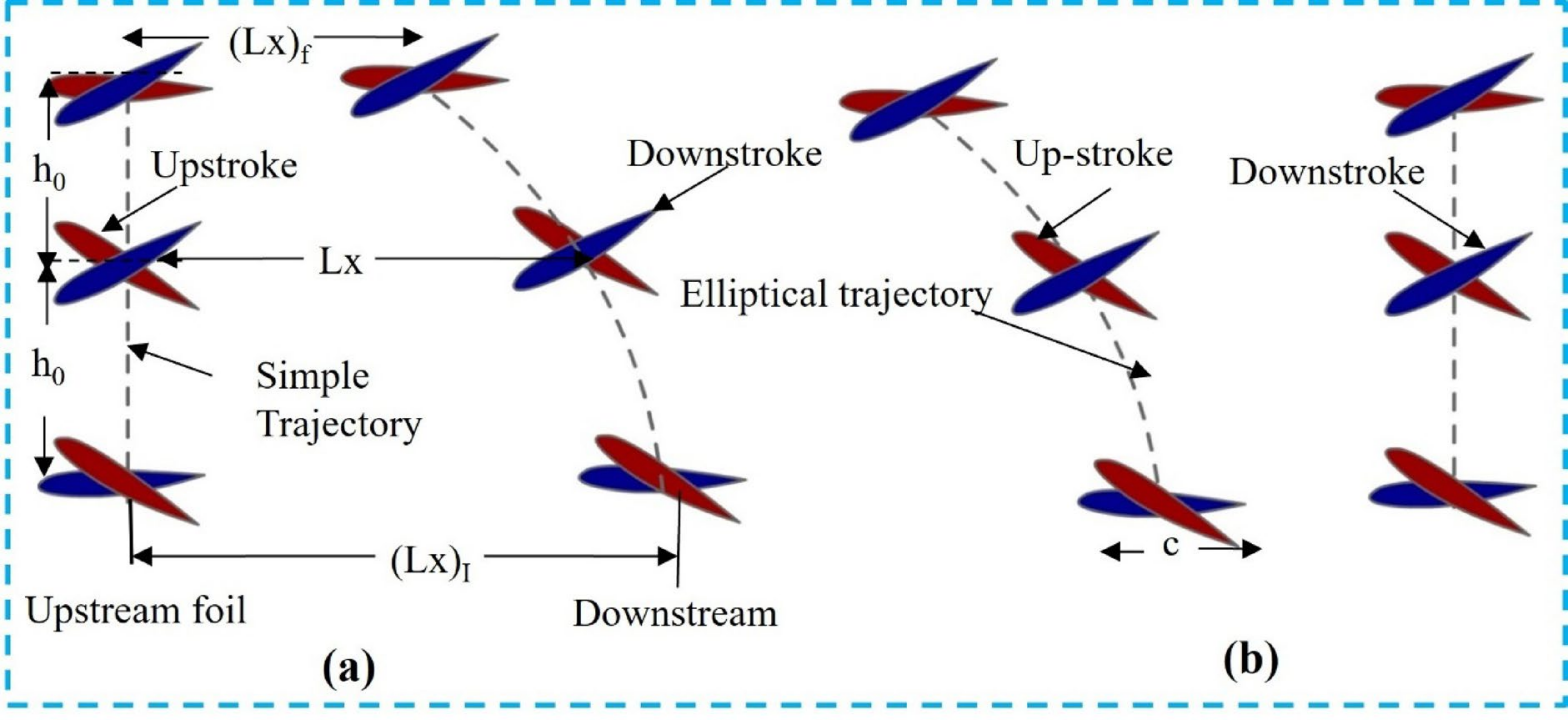
(**a**) Simple flapping motion in a tandem setup (FT-III), (**b**) Elliptical flapping motion in a tandem setup (FT-IV).

At low Reynolds numbers, thin foils are known to perform more propulsively than thicker foils^10^. Furthermore, prior studies show that camber has a minimal effect on foil performance^7^. Thus, in this study, a symmetric, zero-camber foil is used. Figure 1a illustrates a configuration known as the Type-I flapping trajectory (FT-I), in which simple flapping occurs in both the upstream and downstream foils. On the other hand, both foils exhibit elliptical path flapping in the Type-II flapping trajectory (FT-II), as shown in Fig. 1b. The downstream foil in the Type-III flapping trajectory (FT-III), shown in Fig. 2a, flaps elliptically, while the upstream foil flaps simply. In contrast, the Type-IV trajectory (FT-IV) involves simple flapping at the downstream and elliptical flapping at the upstream foil, as shown in Fig. 2b.

An overview of the primary parameters used in the computational model is given in Table 1. For both simple and elliptical trajectories, each foil combines pitching (rotating motion) with heaving (vertical motion). The heave and pitch motions of the foil are represented by Eqs. (1) and (2), respectively.

1 $$h\left(t\right)={h}_{0}{cos}(2\pi ft)$$

2 $$\theta\left(t\right)=-{\theta}_{0}{sin}(2\pi ft+\varphi)$$

The maximum heave amplitude is h_0_, and the maximum pitch amplitude is θ_0_ = 25°. In this case, h(t) and θ(t) stand for the instantaneous heave displacement and pitch angle, respectively. The frequency of flapping is denoted by f, and denotes the phase difference between pitch and heave motions, and c represents the chord length.

The foil gap, defined as ${L}_{x}/c$, corresponds to the relative spacing between the upstream and downstream foils, measured at the mid-stroke (t = T/2), where T is the flapping period. For elliptical flapping trajectories, the forward motion of the foil in the x-direction is given by Eq. (3).

3 $$w\left( t \right) = Sh_{0} \cos (2\pi ft)$$

Where S stands for the elliptical path’s defining element. The energy required for top- and bottom-pitching, travel in the “X” direction, and straight-up-and-down (y-direction) motion is what the flapping foil needs. The effort power required is calculated using Eq. (4), where *F*_*x*_ stands for the X-directional force, *F*_*y*_ stands for the Y-directional force (Fy), and Mt stands for the pitching moments, respectively.

4 $$P=-\frac{1}{T}{\int}_{t}^{t+T}\left[{F}_{x}\left(t\right)\frac{dx\left(t\right)}{dt}+{F}_{y}\left(t\right)\frac{dy\left(t\right)}{dt}+M\left(t\right)\frac{d\theta\left(t\right)}{dt}\right]dt$$

Equations (5), (6), and  (7) are used to calculate the thrust coefficient (*C*_*T*_), power coefficient (*C*_*P*_), and propulsive efficiency (ηT), respectively.

5 $${C}_{T}=\frac{{F}_{T}}{0.5\rho c{U}^{2}}$$

6 $${C}_{P}=\frac{P}{0.5\rho c{U}^{2}}$$

7 $${\eta}_{T}=\frac{{C}_{T}}{{C}_{P}}$$

Where P is the power, and *F*_*T*_ is the thrust force generated. *St* ($St=\frac{2{H}_{0}f}{{U}_{\infty}}$) number is a crucial parameter that affects the flapping foil’s overall performance. Numerous investigations have shown that most ocean and flying species cruise at speeds between 0.2 and 0.5^47,48^. As a result, the *St* number used for this study falls between 0.2 and 0.5.

**Table 1:** The important Parameter of the simulation.

Key parameters	Value
Upstream and downstream foil	NACA0015
*Re*	1173
*St*	0.2, 0.3, 0.4, 0.5
Inter foil gap, *Lx/c*	2, 3, 4, 5
Heaving amplitude, *h*_*0*_	*c*

### 2-D and 3-D computational model

**Fig. 3:**
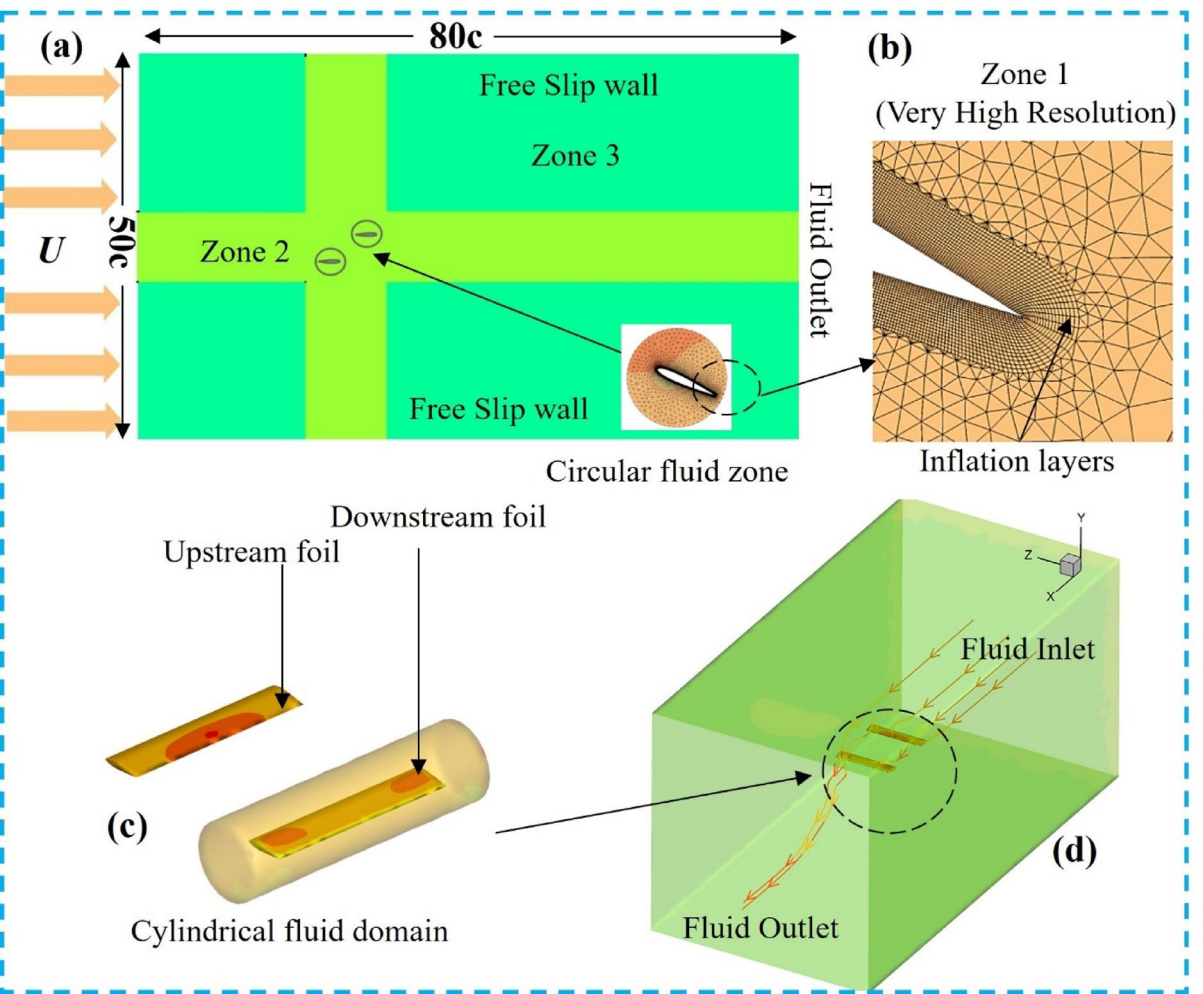
(**a**) Full 2-D computational domain, (**b**) Detailed mesh near the foil tip, (**c**) Cylindrical fluid region, and (**d**) 3-D flow field.

The 2-D and 3-D flapping foil simulations were conducted using ANSYS FLUENT, as illustrated in Fig. 3. Two spherical fluid domains surround the upstream and downstream foils. The inlet and outlet of the fluid are located on opposite sides of a rectangular domain, as illustrated in Fig. 3a. Detailed dimensions of both the circular and rectangular fluid domains are presented in Fig. 3. The circular fluid domain is enclosed by an interference wall. The surface boundaries are free-slip walls or zero-shear-stress boundaries. Both the upstream and downstream foils are surrounded by inflation layers to prevent mesh penetration between the two regions. As shown in Fig. 3b, 20 inflation layers encircle each foil. With a growth rate of 1.2, the first layer height is set to 1 × 10⁻⁴ m. The flow is assumed to be laminar. The circular fluid domain is enclosed by an interference wall. The top and bottom boundaries of the rectangular fluid domain are set with a no-slip condition, or the wall shear stress is near zero.

The 3-D fluid domain includes a cylindrical fluid zone, as illustrated in Fig. 3c. The cylinder has a diameter of 2c and a length of 10c, while the 3-D cross-sectional area measures 50c × 15c, as shown in Fig. 3d. The boundary conditions for the 2-D simulations are similar. Tetrahedral elements are used for 3-D meshing. The distance between the 3-D aerofoils and the inlet boundary is 20c. Equation (1) and Eq. (2) are implemented as User-Defined Functions (UDFs) and integrated into the dynamic mesh according to the flapping trajectory. A velocity–pressure coupled scheme is employed for the transient analysis, and simulations are run until the thrust force converges.

### Grid independence and validation of the current model

**Fig. 4:**
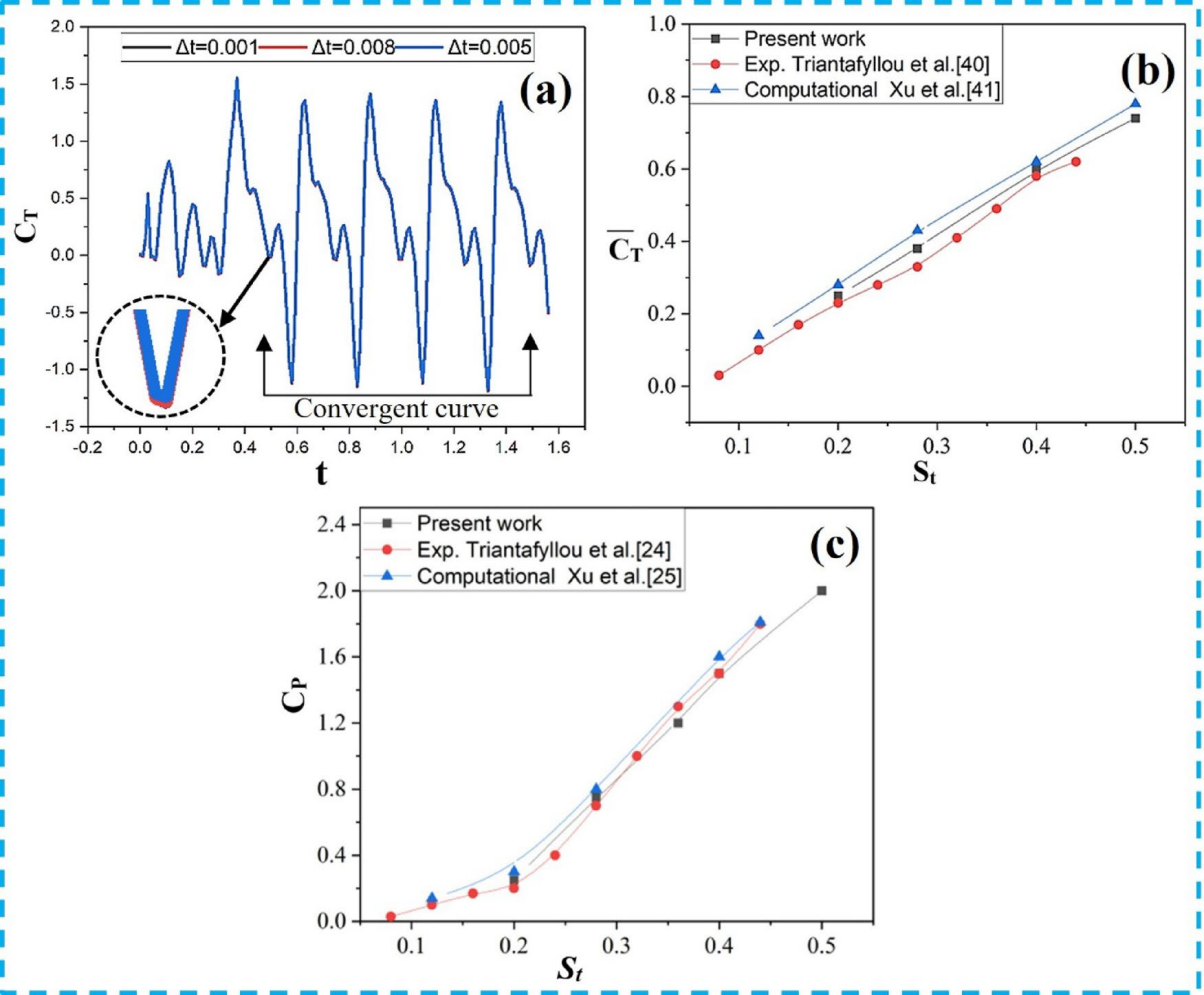
(**a**) Grid convergence results, (**b**) Comparison of thrust curves between the present study and previous work. (c) Power coefficient at various *St.*

Grid and time-step independence were examined using three time-step sets (Δt = 0.001T, 0.005T, and 0.008T), and the results were compared with the experimental data of Triantafyllou et al.^49^ and the numerical results of Xu^50^. The flow was assumed to be laminar, and a second-order upwind scheme was employed for spatial discretization, while a second-order accurate backward implicit scheme was used for time discretization. The selected time steps were t = T/1000, t = 8T/1000, and t = 6T/1000, as listed in Table 2. Figure 4a presents the thrust coefficients over one flapping cycle (T) for different grid configurations considered in the grid-independence study. Figures 4b,c show the thrust and power coefficients of the NACA0015 foil as functions of the Strouhal number. Close agreement with previously reported results is observed, with minor variations attributed to fluid viscosity.

**Table 2:** Mesh independence and validation.

Previous and present study	Grid size	Time step	Mean thrust coefficient
Triatafyllou et al.^49^	160,000	–	0.3966
Xu et al.^50^	Not reported	–	0.4176
Present study (coarse)	188,000	Δt = 0.001T	0.4108
Present study (medium)	263,000	Δt = 0.005T	0.3992
Present study (fine)	365,000	Δt = 0.008T	0.3963

## Results and discussions

### Thrust of a single flapping foil with fishtailed and elliptical flapping trajectory

**Fig. 5:**
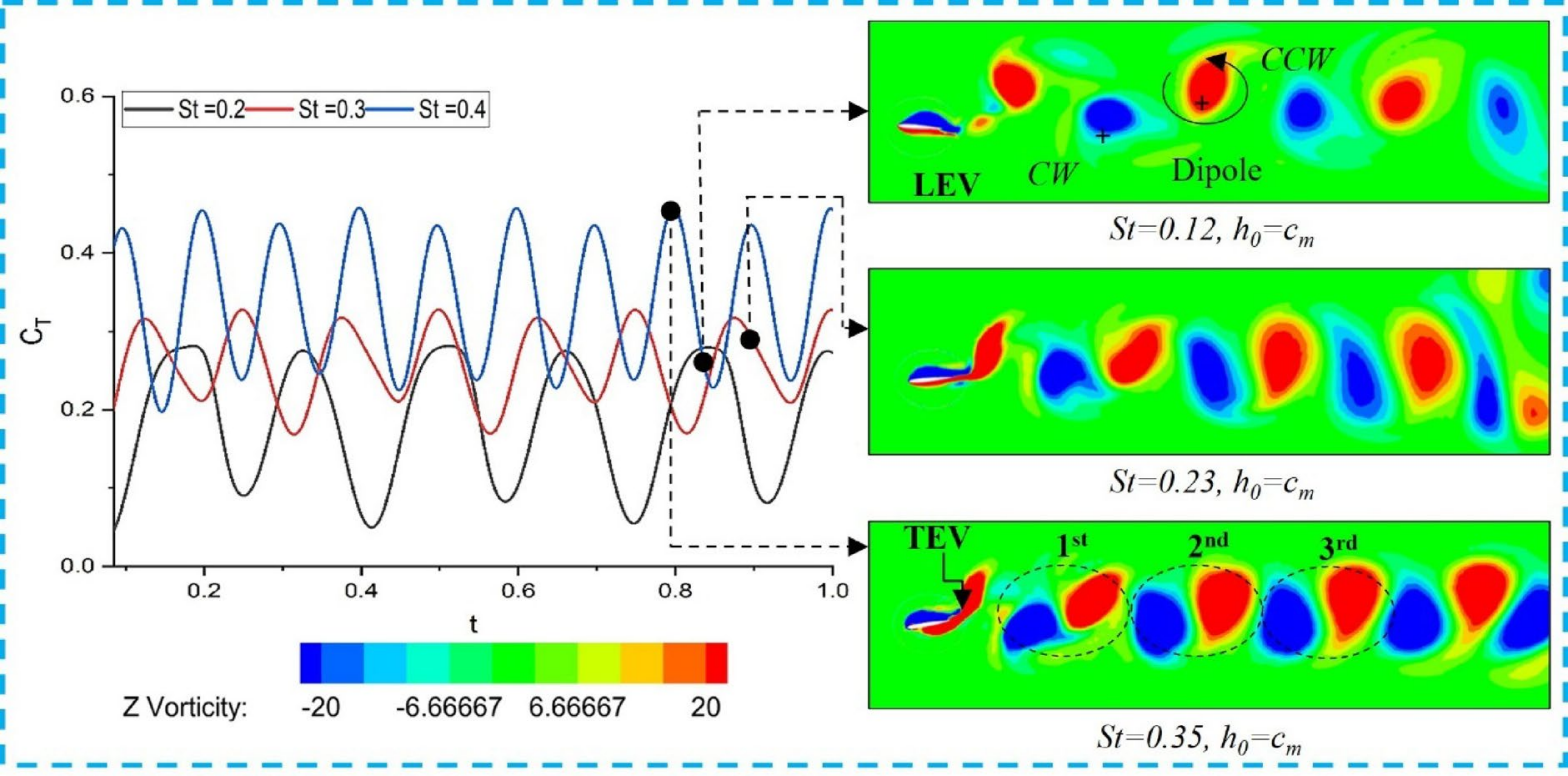
Trailing-edge wake formation produced by a single foil executing a simple flapping motion.

**Fig. 6:**
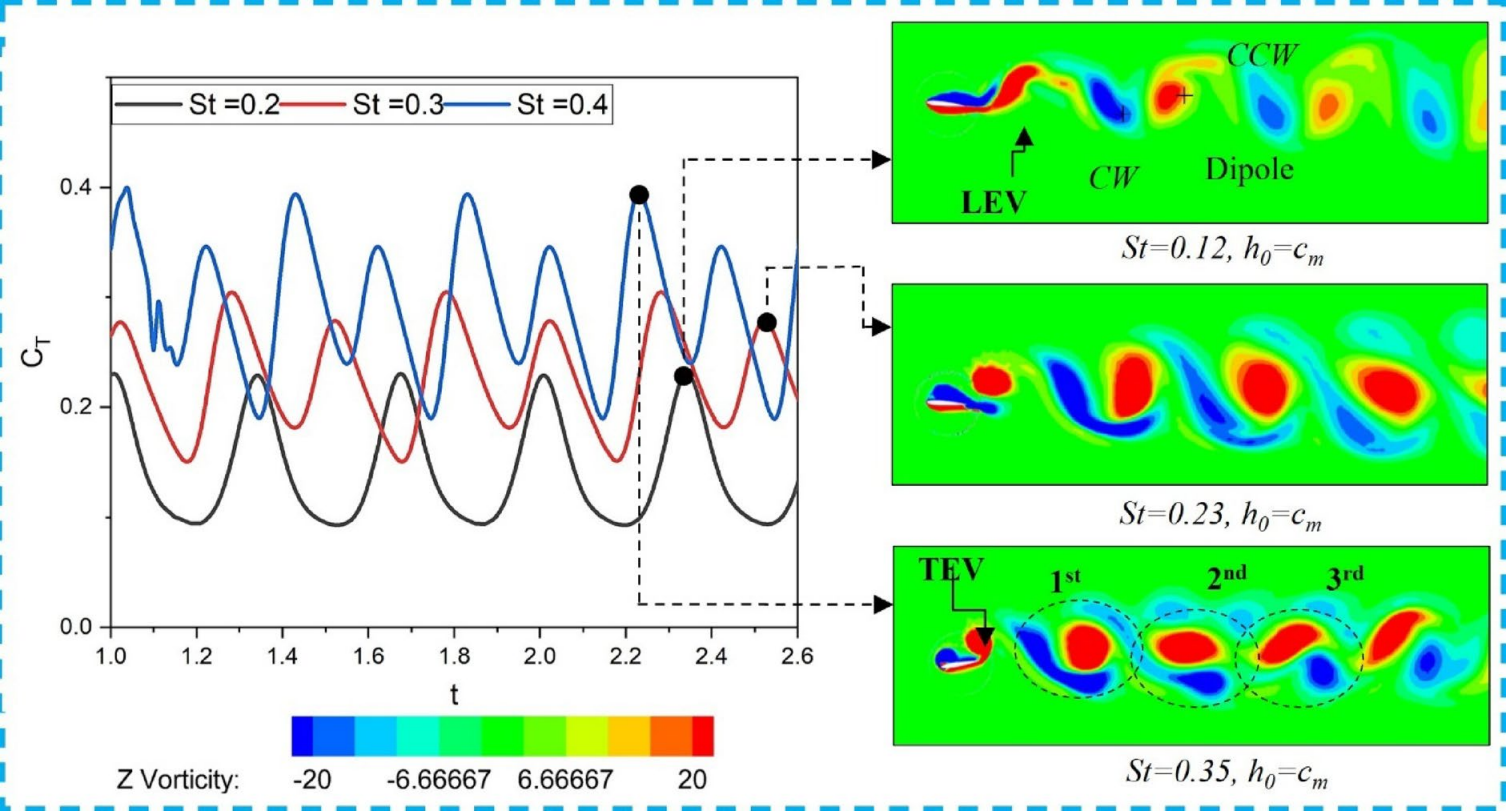
Trailing-edge wake formation produced by a single foil executing elliptical flapping motion.

The flow behavior around the flapping foil is visualized using Z-vorticity contours to understand the thrust-generation mechanism of a single flapping foil. The vorticity contours for simple and elliptical trajectories at various Strouhal numbers (St = 0.2–0.4) are presented in Figs. 5 and 6, respectively. According to Durand^51^, the wake formed at the trailing edge of the foil is influenced by the flapping frequency (St). As the flapping frequency increases, the vortices move closer together (Godoy-Diana et al.^52,53^; Cleaver et al.^54^. The generated vortices form dipole structures aligned horizontally, as shown in Figs. 5 and 6. These dipoles produce a jet in the direction of the fluid flow, which, in turn, induces a reactive thrust that propels the flapping foil forward.

The induced thrust increases with a rising Strouhal number (St), as a higher St generates more dipoles. Consequently, as St increases, the spacing between successive dipoles decreases. According to the Biot–Savart vortex induction law Zheng & Wei^55^, the interaction between vortices is stronger when they are closer together. Hence, the hydrodynamic performance — particularly the induced thrust — of a flapping foil improves at higher St values compared to lower ones. As illustrated in Figs. 5 and 6, for the same St, the first, second, and third induced dipoles in simple flapping appear closer together than those in elliptical flapping. The corresponding induced thrust coefficient for simple flapping is 0.43 (Fig. 5), whereas for elliptical flapping it is approximately 0.40 at the same St (Fig. 6).

### Interaction of vortices in a foil tandem structure

The synergy between the vortices generated by the upstream and downstream foils in a tandem flapping configuration strongly influences the thrust produced by the downstream foil. The Strouhal number (St), the flapping trajectory, and the relative positioning of the foils are the most important factors determining whether this interaction improves or worsens the propulsive performance of the downstream foil. A total of 60 simulations were conducted in this study, varying the foil spacing (Lx/c), the phase angle (Φ) between the foils, and the flapping paths. Three different simulation models were used to examine the interaction between the foils: (i) solo upstream flapping, (ii) solo downstream flapping, and (iii) combined upstream and downstream flapping (FT-I, FT-II, FT-III, FT-IV).

According to all the simulations, the upstream wake can interact with the downstream foil in three different ways:

1. The downstream foil hits the incoming wake from the upstream foil head-on.
2. The downstream foil interacts with the wake on the upper or lower side.
3. It moves back and forth between incoming vortices.

**Fig. 7:**
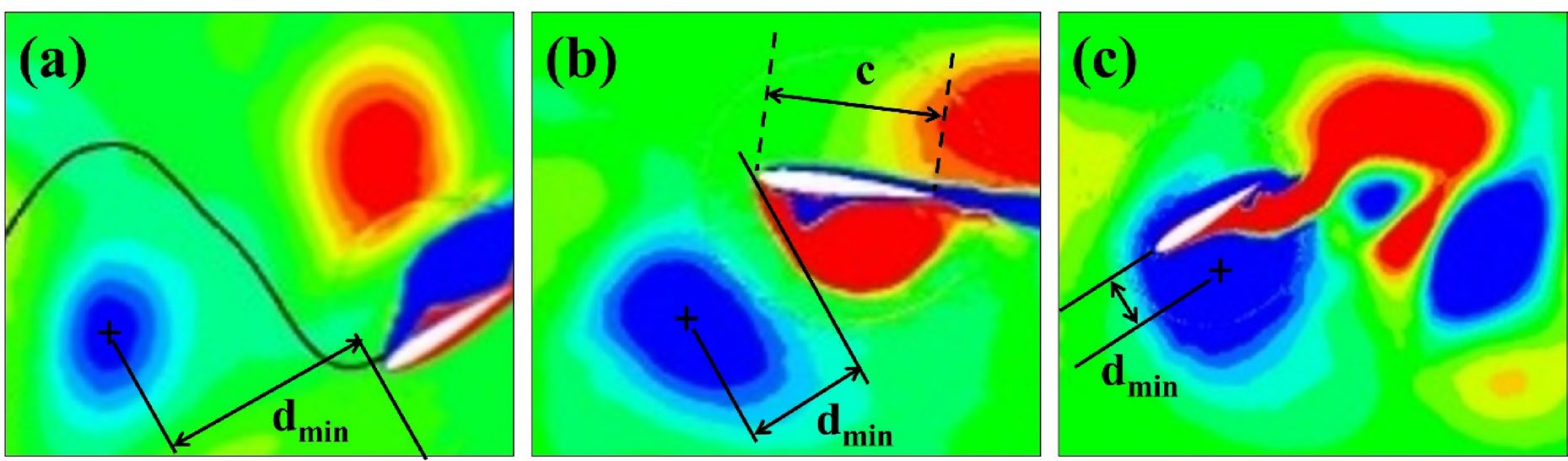
Classification of vortex interaction based on vortex-foil proximity parameter: (**a**) Weak/No-interaction, (**b**) Partial Interaction, (**c**) Direct Collision.

The above interaction is defined using a dimensionless Vortex–foil proximity parameter, Π_*vf*_ = d_min_/c, where ${d}_{min}$ is the minimum distance between the vortex core and the downstream foil leading edge, and *c* is the chord length. Based on the value of Π_*vf*_, the interactions are classified as follows: Weak/No-interaction (Fig. 7a) for Π_*vf*_ ≥ 1.0, Partial Interaction (Fig. 7b) for 0.5 ≤ Π_*vf*_ < 1.0, and Direct Collision (Fig. 7c) for Π_*vf*_ < 0.5, where the vortex core passes within approximately 30% of the foil chord.

**Fig. 8:**
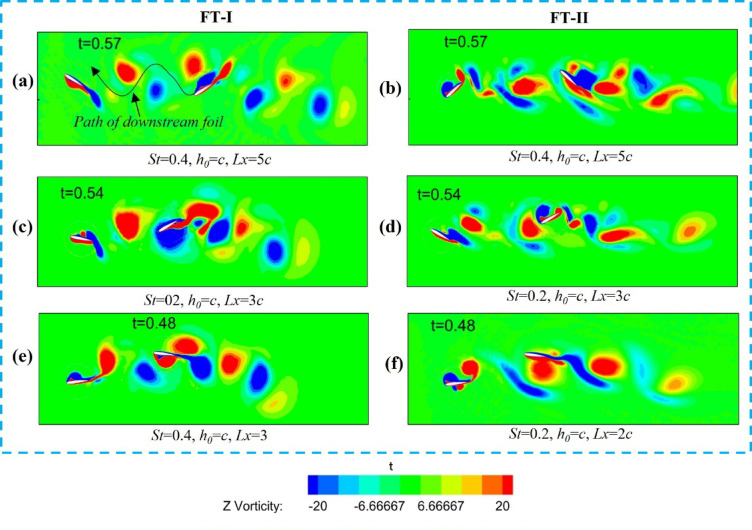
Vorticity contours (− 20 to + 20; red: counter-clockwise, blue: clockwise) for tandem hydrofoils flapping 180° out of phase along FT-I and FT-II trajectories showing different wake–foil interaction regimes: (**a**,** b**) weak/no wake interaction, (**c**, **d**) direct wake-foil collision, and (**e**, **f**) partial interaction based on the proximity parameter (Π_*vf*_).

The manner in which the downstream foil interacts with the upstream wake is used to distinguish different flow regimes. Figure 8 presents vortex contours for tandem hydrofoils undergoing counter-phase (180° out-of-phase) flapping along the FT-I and FT-II trajectories, where Fig. 8a–f represent weak/no-interaction, direct collision, and partial interaction, respectively, as classified by the proximity parameter Π < sub > vf. As shown in Fig. 8a, for *St* = 0.4 and *Lx/c* = 3 (FT-I), the downstream foil passes through the upstream wake without direct collision, corresponding to a weak interaction regime. The wake subsequently rolls up into a dipole structure, resulting in a 38% enhancement in propulsive efficiency of the tandem foil (Table 3, Case-3 for FT-I). Here, η₁ and η₂ denote the propulsive efficiencies of the upstream and downstream foils, respectively.

**Fig. 9:**
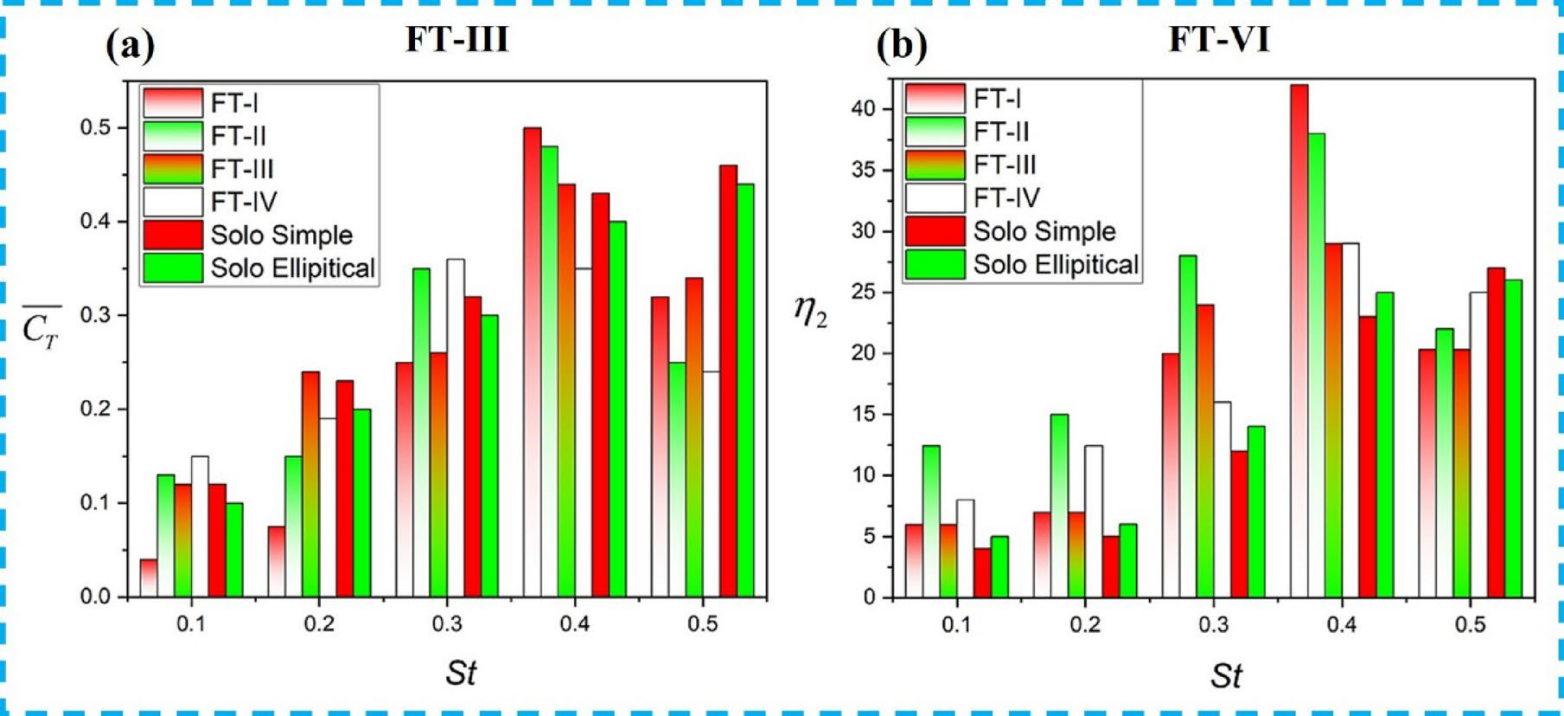
(**a**) Variation of mean thrust with St for the downstream foil in 2-D, (**b**) Propulsive efficiency of the downstream foil under counter-phase flapping in 2-D.

In Case-7 (*St* = 0.4, *Lx/c* = 5), a similar weak-interaction flow pattern is observed for FT-II counter-phase flapping, as illustrated in Fig. 8b, yielding a downstream propulsive efficiency of 24.38%. The corresponding variations of mean thrust coefficient and propulsive efficiency with *St* are shown in Fig. 9a,b, respectively, where both quantities attain their maximum values at *St* = 0.4, with FT-I performing marginally better than FT-II. In contrast, when the downstream foil directly impinges on the upstream wake, as shown in Figs. 8c,d, the interaction is characterized as a direct collision regime. For FT-I at *St* = 0.4 and *Lx/c* = 5 (Fig. 8c), the overall propulsive efficiency drops to 7.2% (Table 3, Case-1).

A similar degradation is observed for FT-II counter-phase flapping in Case-5 (*St* = 0.2, *Lx/c* = 5), illustrated in Fig. 8d, where the downstream propulsive efficiency decreases to 15%. Partial wake interactions, shown in Figs. 8e,f, can either enhance or suppress performance depending on the phase of interaction. As illustrated in Fig. 8e for FT-I and Fig. 8f for FT-II, partial contact between the upstream and downstream wakes occurs for FT-I at *St* = 0.3, *Lx/c* = 3 (Case-2) and *St* = 0.5, *Lx/c* = 3 (Case-4), and for FT-II at *St* = 0.3, *Lx/c* = 3 (Case-6) and *St* = 0.5, *Lx/c* = 2 (Case-8). These partial interactions result in a marginal reduction in induced thrust. Consistent with these observations, Fig. 9a,b confirms that both thrust coefficient and propulsive efficiency peak at *St* = 0.4 for both flapping modes, with FT-I consistently exhibiting slightly higher efficiency than FT-II.

**Table 3:** 2-D and 3-D flapping outcome [h_0_/c = 1] for φ = 180⁰ phase angle following FT-I, FT-II, FT-III, and FT-IV flapping.

Case	*St*	*L*_*x*_ */c*	α(º)	*θ*_*1*_	*θ*_*2*_	Π_vf_	2-D		3-D	
							*η*_*1*_	*η*_*2*_	*η*_*3*_	*η*_*4*_
FT-I flapping										
1	0.2	5	10	75	75	0.1	7	7.2	6.9	6.98
2	0.3	4	8	53	65	0.3	20	20	20.1	20.14
3	0.4	3	7	56	73	1.2	27	42	27.2	42.65
4	0.5	5	7	56	73	0.28	20.32	20.32	19.9	20.71
FT-II flapping										
5	0.2	2	10	75	75	0.15	7	15	7.12	14.69
6	0.5	3	8	53	65	4	24	28	24.16	28.77
7	0.4	5	7	56	73	0.8	29	38	30.6	37.21
8	0.3	2	7	56	73	0.3	20.32	22.7	20.32	23
FT-III flapping										
9	0.1	2	10	75	75	0.15	7.1	6.96	6.98	12.69
10	0.5	3	8	53	65	0.8	19.98	24	20	24.77
11	0.4	5	7	56	73	0.9	26.72	29.12	26.8	29
12	0.3	3	7	56	73	0.6	21	20.32	20.32	21
FT-IV flapping										
13	0.1	2	10	75	75	0.11	6.78	12.43	6.55	12
14	0.5	3	8	53	65	0.5	23.74	16	23.68	16.63
15	0.4	5	7	56	73	1.1	28.75	31	27.63	31.74
16	0.3	3	7	56	73	0.7	19	25	18.75	25.21

Table 3 Parameters: α = effective angle of attack (°); θ₁ = pitch angle of the upstream foil (°); θ₂ = pitch angle of the downstream foil (°); φ = phase difference (radians); St = Strouhal number (dimensionless); η₁, η₃ = propulsive efficiency of the upstream foil in 2-D and 3-D, respectively (%); η₂, η₄ = propulsive efficiency of the downstream foil in 2-D and 3-D, respectively (%).

**Fig. 10:**
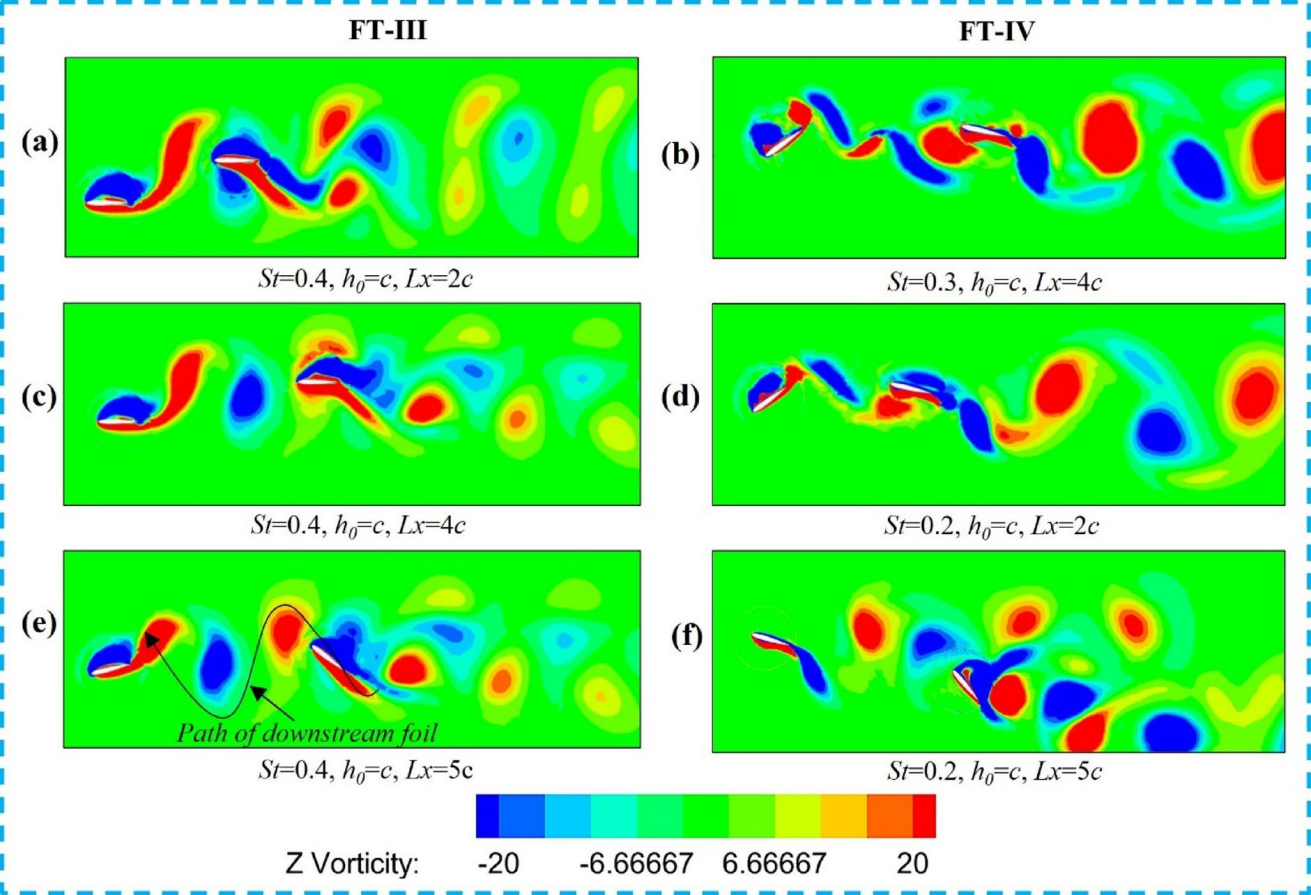
Vorticity contours (− 20 to + 20; red: counter-clockwise, blue: clockwise) for tandem hydrofoils flapping 180° out of phase along FT-III and FT-IV trajectories showing different wake–foil interaction regimes: (**a**, **b**) weak/no wake interaction, (**c**, **d**) direct wake-foil collision, and (**e**, **f**) partial interaction based on the proximity parameter (Π_*vf*_).

For the other two tandem flapping arrangements, Fig. 10a,b,e,f illustrate the wake–foil interaction regimes for FT-III and FT-IV counter-phase flapping. In the weak-interaction regime, Fig. 10e shows that at St = 0.4 and Lx/c = 5 (FT-III flapping), the downstream foil passes through the wake shed by the upstream foil without collision (Π*vf* = 0.9). The wake subsequently rolls up into a dipole structure, enhancing the tandem foil’s overall propulsive efficiency to 29% (Table 3, Case-11, FT-III). A similar weak-interaction flow pattern is observed for FT-IV counter-flapping in Case-15 (St = 0.4, Lx/c = 5), as shown in Fig. 10f, resulting in a downstream propulsive efficiency of 31%. As evident from Fig. 9a,b, both the propulsive efficiency and thrust coefficient attain their maximum values at St = 0.4, and at this Strouhal number, FT-III and FT-IV exhibit nearly comparable performance.

In contrast, direct wake–foil collision is observed for FT-III flapping at St = 0.4 and Lx/c = 5, as shown in Fig. 10c,d, where the downstream foil directly impinges on the incoming wake (Π_*vf*_ = 0.15). This interaction reduces the overall propulsive efficiency of the tandem foil by approximately 7% (Table 3, Case-9, FT-III). A similar collision-dominated flow regime is observed for FT-IV counter-flapping in Case-13 (St = 0.2, L*x/c* = 5), leading to a downstream propulsive efficiency of only 12%.

Partial wake interactions, characterized by intermediate values of the proximity parameter, are shown in Fig. 10e,f. For FT-III flapping, partial interactions occur at St = 0.5, Lx/c = 3 (Case-10, Π_*vf*_ = 0.8) and at St = 0.3, Lx/c = 3 (Case-12, Π_*vf*_ = 0.6). For FT-IV flapping, similar partial interactions are observed at St = 0.3, Lx/c = 3 (Case-14) and St = 0.5, Lx/c = 2 (Case-16), both with Π<_*vf*_ ≈ 0.5. These partial interactions result in a slight reduction in thrust. Consistent with these observations, Fig. 9a,b confirm that both thrust coefficient and propulsive efficiency peak at St = 0.4 for both flapping modes, with FT-III exhibiting marginally higher efficiency than FT-IV.

**Fig. 11:**
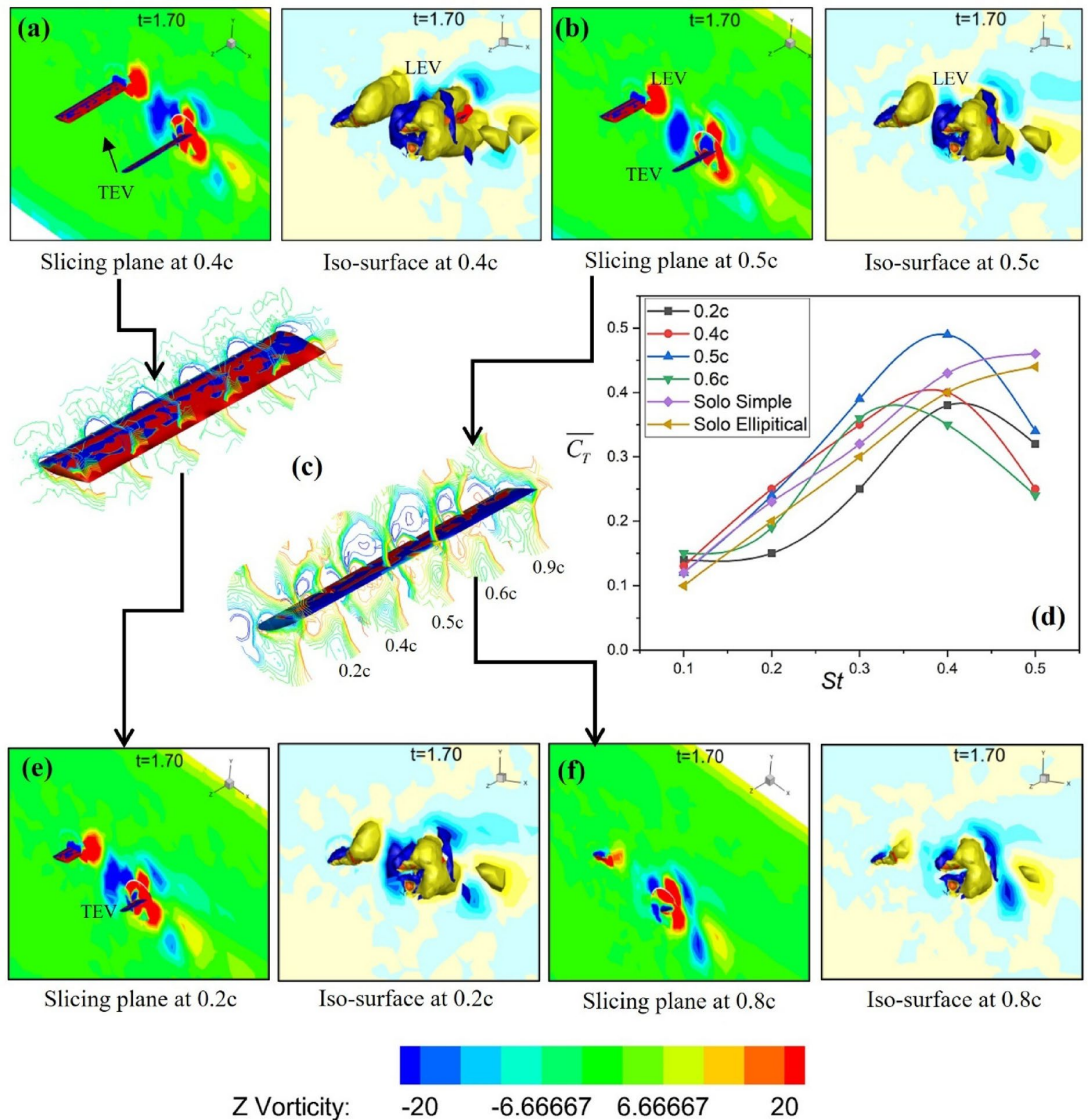
Counter-phase FT-I flapping: (**a**) Slice plane and iso-surface at 0.4c, (**b**) Slice plane and iso-surface at 0.5c, (**c**) Upstream and downstream foils with sectional slicing, (**d**) Variation of induced thrust spanwise, (**e**) Slice plane and iso-surface at 0.2c, and (**f**) Slice plane and iso-surface at 0.8c.

The 3-D simulation results are further compared with the efficiencies obtained in 2-D flapping. Among the different trajectories, FT-I flapping demonstrates the highest efficiency; hence, it is analyzed in greater detail along the span using vortex field visualization. The Q value for the iso-surface in the present study is 8.12 × 10⁴ s^-2^. Figure 11a–f illustrates the net force acting on the tandem foil during the downstroke for the FT-I trajectory. The force increases notably in the range from 0.4c to 0.6c. Figure 11c shows the foil positions at four chordwise locations (0.2c, 0.4c, 0.6c, and 0.8c) at different flapping times, while Fig. 11b presents the upstream foil’s leading-edge vortex (LEV) position relative to the downstream foil along the chord. A stronger LEV is observed at mid-downstroke around 0.5c due to the high angle of attack (AOA).

The flow visualizations in Fig. 11a,b,e,f illustrate wake vortex slicing at chordwise locations of 0.4c, 0.5c, 0.2c, and 0.8c, respectively, for counter-phase FT-I flapping. The counterclockwise (CCW) vortex shed from the upstream foil interacts with the upper surface of the downstream foil, thereby enhancing thrust and propulsive efficiency. Figure 11c shows the upstream and downstream foils with sectional slicing, while Fig. 11d presents the spanwise variation of induced thrust, demonstrating superior performance at St = 0.4 compared to other Strouhal numbers.

**Fig. 12:**
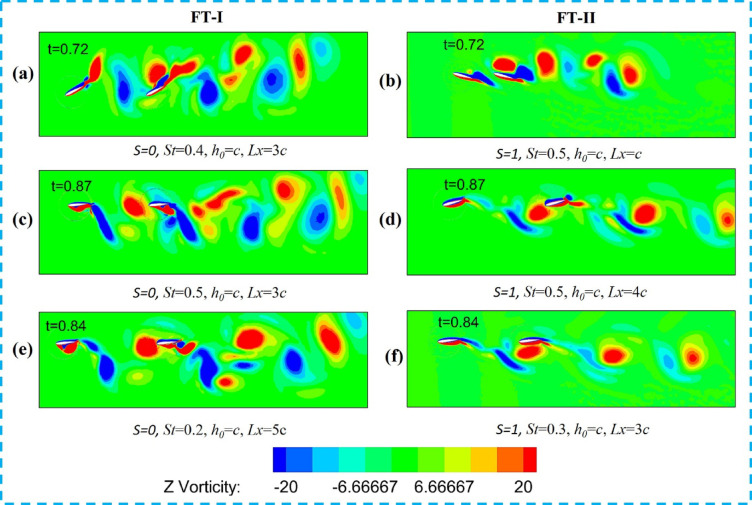
Z-vorticity contours (− 20 to + 20; red: counter-clockwise, blue: clockwise) for upstream and downstream foils under in-phase (0°) flapping along FT-I and FT-II trajectories showing different wake–foil interaction regimes: (**a**, **b**) weak/no wake interaction, (**c**, **d**) direct wake-foil collision, and (**e**, **f**) partial interaction based on the proximity parameter (Π_*vf*_).

The Z-vorticity distribution of the upstream and downstream foils during in-phase flapping for the FT-I and FT-II trajectories is shown in Fig. 12. Similar to counter-phase flapping, the in-phase cases are also classified using the vortex-foil proximity parameter (Π_*vf*_), which indicates whether the downstream foil collides with the wake shed by the upstream foil. As shown in Fig. 12a,b, for St = 0.4, L*x/c* = 3 (FT-I) and St = 0.4, L*x/c* = 2 (FT-II), the downstream foil passes through the upstream wake without collision, corresponding to Π_*vf*_ = 1.3 and Π_*vf*_ = 1.0, respectively. The wake vortices subsequently merge to form a coherent dipole, enhancing the overall propulsive efficiency of the tandem foil. These cases correspond to Table 4 (FT-I case 4 and FT-II case 7).

**Table 4:** 2D tandem results for NACA 0012 [*h*_*0*_/c = 1] for in-phase flapping.

Case	St		L_x_/c	α(º)	θ_1_	θ_2_	Π_vf_	2-D		3-D	
								η_1_	η_2_	η_3_	η_4_
Flapping trajectory FT-I											
1	0.2		5	10	75	75	0.4	16.83	20	15.69	20.23
2	0.3		2	7	56	73	0.98	23.14	28	22.77	28.34
3	0.4		4	8	53	65	1.1	30.67	30	31	29.65
4	0.5		3	7	56	73	1.3	33.5	29	33.17	29.65
Flapping trajectory FT-II											
5	0.2		2	10	75	75	0.85	12.69	30	12	31
6	0.3		2	7	56	73	1.2	18.77	36	18	35.13
7	0.4		3	8	53	65	1	23.17	35.12	23	36
8	0.5		4	7	56	73	0.45	25	28	25.13	28.21
Flapping trajectory FT-III											
9	0.2		2	10	75	75	0.65	12.69	22	12	22.34
10	0.3		2	7	56	73	0.7	18.77	25	16	25.21
11	0.4		3	8	53	65	0.8	23.17	28	23	27.65
12	0.4		4	7	56	73	0.85	25	29	25	29.42
Flapping trajectory FT-IV											
13	0.2		2	10	75	75	1.1	12.69	35	13	35.16
14	0.3		2	7	56	73	1.2	18.77	39.13	18	40
15	0.4		3	8	53	65	1.4	23.17	47	23	46.15
16	0.5		4	7	56	73	0.85	25	29	25	29

**Fig. 13:**
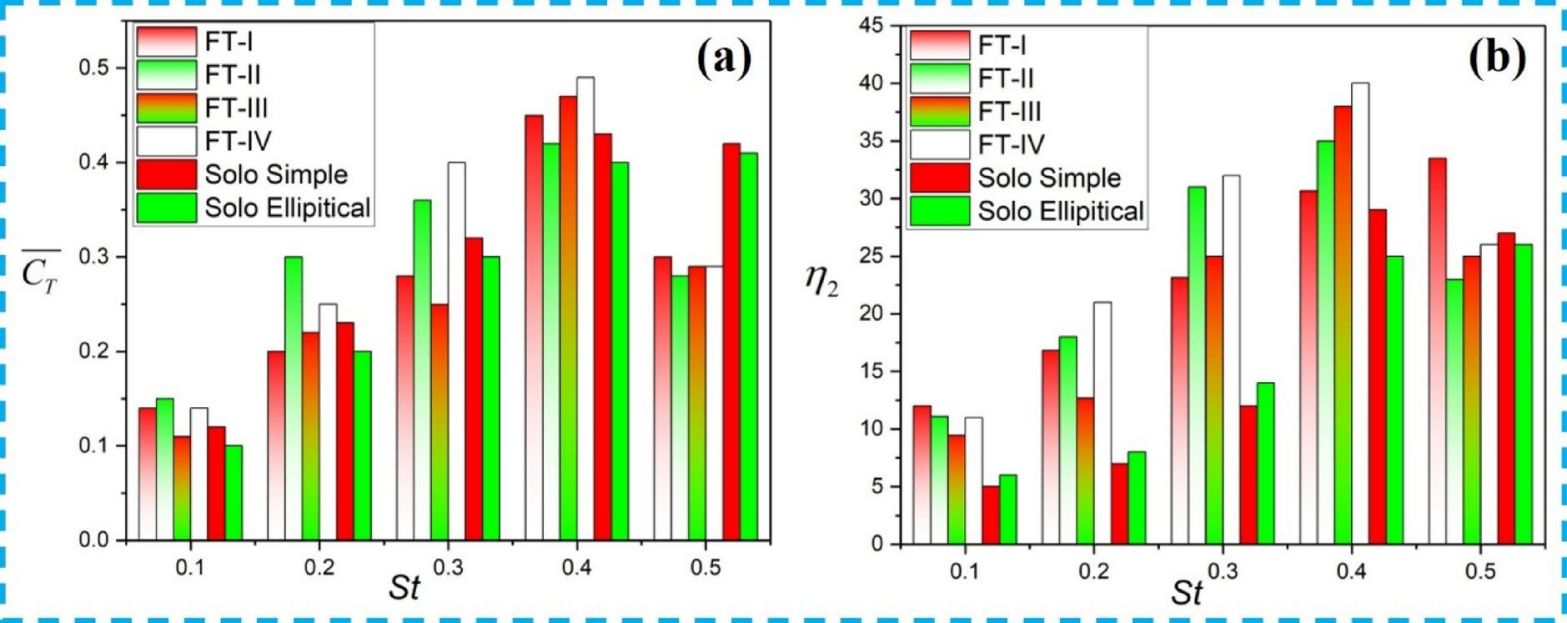
(**a**) Variation of mean thrust with St for the downstream foil in 2-D, (**b**) Propulsive efficiency of the downstream foil under in-phase flapping in 2-D.

In contrast, Fig. 12c,d illustrate cases of direct collision for St = 0.5, L*x/c* = 3 (FT-I) and St = 0.5, L*x/c* = 4 (FT-II), where the downstream foil directly interacts with the upstream wake (Π_*vf*_ < 0.5). This interaction disrupts dipole formation, leading to reduced induced thrust, as summarized in Table 4 (FT-I case 1 and FT-II case 8). Consequently, the thrust obtained is lower than that of a solo flapping foil, as shown in Fig. 13a. Partial wake interactions are observed in Fig. 12e,f for FT-I flapping at (St = 0.2, L*x/c* = 5) and (St = 0.4, L*x/c* = 4), and for FT-II flapping at (St = 0.5, L*x/c* = 2) and (St = 0.5, L*x/c* = 3). These cases result in a marginal reduction in induced thrust and are summarized in Table 4 (FT-I cases 1 and 3, and FT-II cases 5 and 7). From Figs. 13a,b, it is evident that both the thrust coefficient and propulsive efficiency reach their maximum values at St = 0.4 for elliptical flapping under both FT-I and FT-II trajectories.

**Fig. 14:**
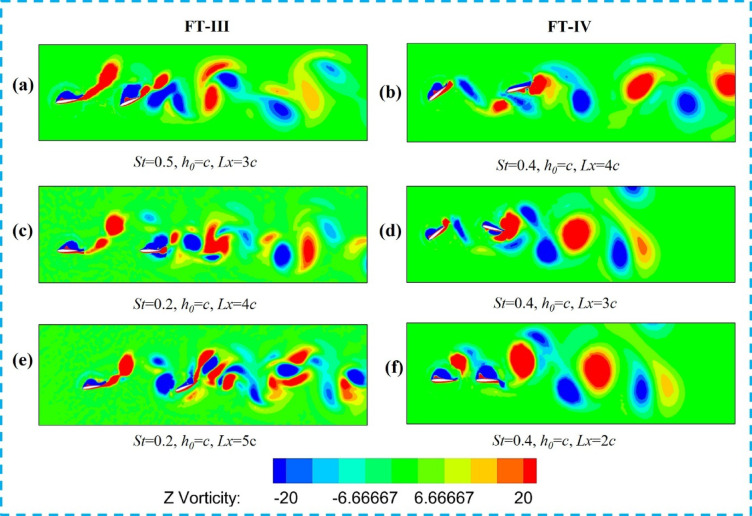
Vortex contour plots (− 20 to + 20; red: counter-clockwise, blue: clockwise) for upstream and downstream foils under in-phase (0°) flapping along FT-III and FT-IV trajectories showing different wake–foil interaction regimes: (**a**, **b**) weak/no-wake interaction, (**c**, **d**) direct wake-foil collision, and (**e**, **f**) partial interaction based on the proximity parameter (Π_*vf*_).

For the FT-III and FT-IV trajectories, the corresponding in-phase wake interaction patterns are illustrated in Fig. 14. As seen in Fig. 14a,b, for FT-III flapping at St = 0.5, L*x/c* = 4, the downstream foil passes through the upstream wake without collision, leading to dipole formation and an enhanced propulsive efficiency of 47%, corresponding to Table 4 (FT-III case 11). Similar trends are observed for FT-IV flapping: weak/no-interaction cases yield higher efficiency, whereas direct collision and partial interaction cases shown in Fig. 14c,f result in reduced thrust. Overall, Figs. 13a,b indicate that for all flapping trajectories and interaction modes, the propulsive efficiency and thrust coefficient peak at St = 0.4, with FT-III and FT-IV exhibiting comparable efficiency levels.

**Fig. 15:**
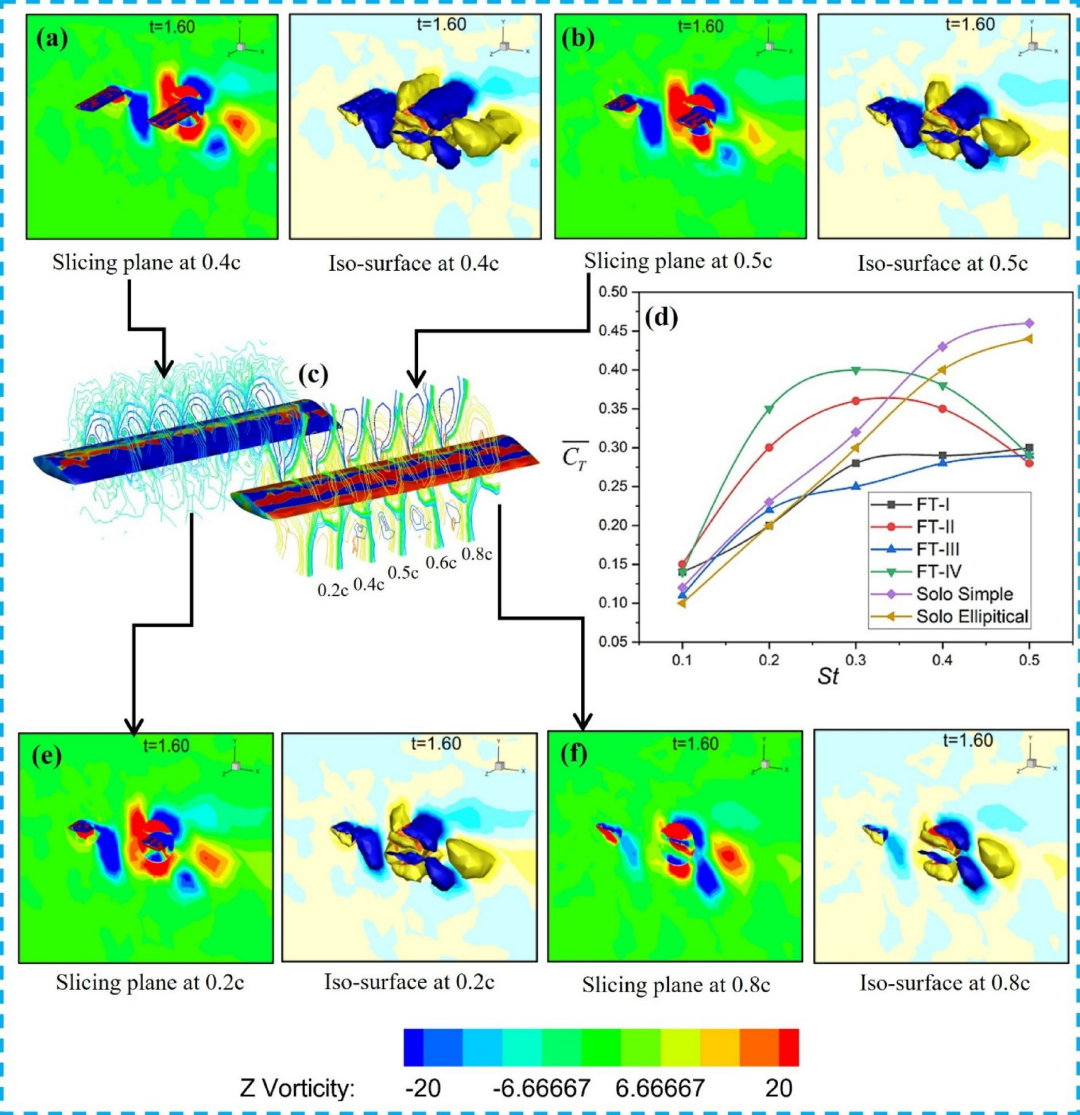
Type-I flapping in in-phase mode (Three-dimensional vortex structures visualized using Q-criterion iso-surfaces (Q = 0.5): (**a**) slicing a plane with an iso-surface at 0.4c, (**b**) slicing a plane with an iso-surface at 0.5c, (**c**) Sliced views of upstream and downstream foils, (**d**) Induced thrust varying spanwise, (**e**) Slicing plane with iso-surface at 0.2c, (**f**) Slicing plane with iso-surface at 0.8c.

The results of the 2D investigation were further examined using three-dimensional flapping simulations to validate the key findings. As shown in Fig. 15, for Type-I flapping in in-phase mode, the downstream foil at L_x_/c = 4 exhibits the highest thrust efficiency (47%; see Table 4). The three-dimensional vortex structures, visualized using Q-criterion iso-surfaces (Q = 0.5), reveal the detailed mechanisms governing wake interactions and performance. Figure 15a,b,e,f illustrate vortex iso-surface slicing at 0.4c, 0.5c, 0.2c, and 0.8c, respectively, highlighting the spanwise evolution of coherent vortical structures. Figure 15c presents sliced views of the upstream and downstream foils, while Fig. 15d shows the spanwise variation of induced thrust. Notably, at mid-span sections, a counter-clockwise (CCW) vortex forms near the upper surface of the downstream foil, promoting a favorable pressure distribution and enhanced thrust. However, for higher Strouhal numbers (St = 0.4–0.5), adverse vortex–foil interactions, including direct wake impingement, are observed in Fig. 15, leading to reduced tandem-foil performance compared to solo flapping.

## Discussion

As it has been shown in the current work, the process of the vortex interaction in the tandem flapping-foil arrangement is quite sensitive to three main parameters: Strouhal number (St), phase angle (Φ), inter-foil spacing (L_x_/c). The highest efficiency is found for the FT-I trajectory with St = 0.4, L_x_/c = 3, and Φ = 180° and the resulting downstream-foil propulsive efficiency is 42% (equivalent to 56% better than the solo flapping at the same St). This improvement is attributed to positive interactions between the vortex and the foil, with the downstream foil between the upstream vortices essentially absorbing energy from the organized wake.

Vortex dynamics can explain the physical mechanism behind thrust augmentation. When encountering a counter-rotating vortex pair shed by the upstream foil, the downstream foil experiences an induced velocity field that speeds up the flow over its surfaces. This interaction increases the effective angle of attack, producing extra lift-based thrust. This mechanism is most effective when the vortex spacing is well matched with the heaving amplitude of the downstream foil.

Direct vortex–foil collision (Π_vf_ < 0.5), however, results in thrust degradation due to flow separation and adverse pressure gradients on the downstream foil. This is seen when the inter-foil spacing is either excessively large (L_x_/c = 5) or when the phase relationship is misaligned, causing the downstream foil to collide directly with incoming vortices rather than passing between them. Three-dimensional analysis also shows that finite-span effects significantly modify the vortex-interaction dynamics predicted by 2-D simulations. Nevertheless, the qualitative trends and optimal parameter combinations identified in 2-D remain valid in 3-D, indicating that 2-D analysis provides useful design guidance for moderate-to-high aspect ratio configurations (AR > 4) commonly used in bio-inspired propulsors.

The results have direct implications for the design of soft-robotic swimmers and bio-inspired underwater vehicles. The identified optimal configurations (FT-I and FT-III at St = 0.4 with appropriate phase and spacing) can be integrated into multi-foil propulsion systems to achieve substantial efficiency gains. For example, a tandem-foil autonomous underwater vehicle operating at cruise conditions would consume 35% less power than a single-foil underwater vehicle by exploiting favorable vortex interactions.

Although this study provides valuable insights into tandem flapping-foil hydrodynamics, there are limitations that should be acknowledged. Simulations were performed at Re = 1173, representative of small-scale bio-inspired swimmers (body length L ≈ 10 cm, speed U ≈ 0.1 m/s), but considerably lower than those of full-scale underwater vehicles (Re ≈ 10⁴–10⁶). At Re = 1173, the flow remains laminar throughout the flapping cycle, whereas at higher Reynolds numbers it transitions to turbulence, which can significantly alter the process of energy dissipation, vortex dynamics, and wake structure.

## Conclusions

In this work, the hydrodynamic performance of tandem flapping foils on simple and elliptical trajectories at Re = 1173 is numerically investigated. Four different tandem configurations (FT-I, FT-II, FT-III, and FT-IV) are examined by varying the flapping route, the Strouhal number (St), and the phase angle (Φ = 0° and 180°). The largest impact on the thrust generated by a single foil is the wake dynamics at the trailing edge. Thrust is increased by the simple flapping route, which creates a greater reverse Kármán vortex street than the elliptical motion. Due to stronger vortex interactions and closer dipole spacing, a higher Strouhal number causes further increases in thrust.

In tandem configurations, the performance of the downstream foil is strongly influenced by its interactions with the vortices of the upstream foil. These interactions are categorized according to the vortex-foil proximity parameter. For St = 0.4, the FT-I configuration (Φ = 180°) demonstrated the highest induced thrust, while FT-III (Φ = 0°) showed comparable efficiency under in-phase motion. The axial spacing (Lx/c) and the interaction between the vortices have a significant impact on the propulsive efficiency of the downstream foil. However, the downstream position has little effect on the upstream foil.

### Key highlights of findings

- Simple flapping produces stronger reverse Kármán vortices → higher induced thrust.
- The higher Strouhal number (St) enhances vortex interaction → improved thrust generation.
- FT-I (Φ = 180°) achieves the peak induced thrust at St = 0.4.
- FT-III (Φ = 0°) performs efficiently with synchronized and in-phase motion.
- Downstream foil efficiency depends on axial spacing and wake–vortex interaction.
- Optimized phase synchronization and trajectory design can significantly improve hydrodynamic performance, giving designers ideas for bio-inspired marine propulsion and energy-efficient underwater vehicles.

## Data Availability

The data used to support the findings of this study are included within the article.
